# Medical and Surgical Memoirs

**Published:** 1871-06

**Authors:** 


PREPARING FOR THE PRESS.

---

Medical and Surgical Memoirs. By Joseph Jones, M.D., Professor of Chemistry, Medical Department, University of Louisiana.

This work will embrace the investigations of fifteen years into the Causes, Geographical Distribution, Natural History and Treatment of Intermittent, Remittent and Congestive Malarial Fevers, Yellow Fever, Typhoid and Typhus Fevers, Small-Pox, Spurious Vaccination, Measles, Pneumonia, Diarrhœa, Dysentery, Scurvy, Tetanus, Cerebro-Spinal-Meningitis, Diseases supervening upon Gun-Shot Wounds, Pyæmia, Hospital Gangrene, Erysipelas, etc.

The results of the investigation of the Diseases of the Confederate Army, during the American Civil War, 1861-1865, will occupy a prominent portion of the Work.

These investigations have been prosecuted unremittingly during the past 15 years; and the author proposes to lay the results before the Medical Profession, as soon as a sufficient number of subscribers have been obtained.

Physicians and others desiring to become subscribers, will please forward their names to

Joseph Jones, M.D., Glass Box 1542, New Orleans, La.

